# Assessing the socio-cognitive determinants of personal protective equipment uses among domestic waste collectors in the Ho municipality, Ghana: A cross-sectional study

**DOI:** 10.1371/journal.pone.0334542

**Published:** 2025-11-04

**Authors:** Samuel Yaw Lissah, Martin Amogre Ayanore, John K. Krugu, Matilda Aberese-Ako, Robert A.C. Ruiter

**Affiliations:** 1 Department of Work and Social Psychology, Faculty of Psychology and Neuroscience, Maastricht University, Maryland Maastricht, The Netherlands; 2 Department of Agricultural Sciences and Technology, Faculty of Applied Sciences and Technology, Ho Technical University, Ho, Volta Region, Ghana; 3 Department of Health Policy Planning and Management, School of Public Health, University of Health and Allied Sciences, Ho, Volta Region, Ghana; 4 KIT Royal Tropical Institute, Amsterdam, The Netherlands; 5 Institute of Health Research, University of Health and Allied Sciences, Ho, Volta Region, Ghana; University of California Davis, UNITED STATES OF AMERICA

## Abstract

Domestic waste collectors (DWCs) are exposed to occupational safety and health related morbidities and mortalities globally due to the non-use, improper use, and non-availability of personal protective equipment (PPE) in their jobs which endangers DWCs’ lives, safety, and well-being. The present study investigated the extent to which socio-cognitive determinants predicted PPE use among DWCs in the Ho municipality in the Volta Region in Ghana. A quantitative cross-sectional survey was conducted among DWCs (n = 344) in the Ho Municipality of Ghana to assess the socio-cognitive determinants of PPE use. The questionnaire consisted of 107 items that were informed by a literature review in previous qualitative research, and two theoretical frameworks explaining behavior (i.e., the Health Belief Model (HBM) and Reasoned Action Approach (RAA) and measured constructs such as perceived severity and susceptibility of work-related health risks, perceived benefits, and barriers, perceived norm, and self-efficacy towards PPE use. Partial least squares structural equation modeling (PLS-SEM) was used to evaluate the structural model describing the relationship between the socio-cognitive determinants and intention to use PPE, which was the main outcome measure. The integrated model explained 67% of the variance in PPE-use intention. Intention to use PPE was significantly positively and directly influenced by attitude (β = 0.174, p < 0.001), indicated cues to action (β = 0.500, p < 0.001), perceived rule enforcement by the management (β = 0.114, p < 0.05), and self-efficacy (β = 0.199, p < 0.01). The direct effect of subjective norms on intention to use PPE was not significant (β = 0.040, p = 0.396). Attitude in turn was significantly predicted by perceived severity (β = 0.244, p < 0.001), perceived benefits (β = 0.209, p < 0.01), and behavioral beliefs (β = 0.342, p < 0.001), whereas perceived barriers were significantly associated with self-efficacy (β = 0.377, p < 0.001). In conclusion, the current study successfully expanded the utility of HBM and RAA in assessing the socio-cognitive determinants of PPE use among DWCs in a developing economy. Thus, the findings highlight the combined influence of individual beliefs and organizational enforcement on DWCs’ motivation to use PPE. Interventions should pair hazard‑communication and self‑efficacy training with strict managerial enforcement to strengthen PPE compliance.

## Introduction

Globally, domestic waste collectors (DWCs) face high level of occupational health risks including exposure to pathogens, sharp objects, and toxic substances, which are hazards that can lead to work-related injuries and diseases [1–3]. For example, an estimated two million DWCs suffer from health-related complications annually [1,3]. According to the International Labor Organization (ILO), over 2.9 million workers die annually from work-related injuries and diseases, with a large percentage of these deaths taking place in hazardous sectors like waste management [4]. About 34% and 13% of occupational accidents are attributable to the non-use and inappropriate use of personal protective equipment (PPE) [4,5]. DWCs are exposed to numerous hazards that pose significant health risks, increasing their likelihood of adverse health outcomes such as occupational diseases, injuries, or disabilities [4,6]. The lack of knowledge and skills on proper waste handling, lack of education and awareness on occupational health risks, non-availability of PPE, inappropriate PPE use, and non-use of PPE are among the reasons that exacerbate health risks among DWCs [7–10]. Evidence suggests that PPE such as ear protection devices, gloves, wellington boots, face, and nose masks can reduce hazards and work-related risks in unsafe work environments [7,11,12].

In many parts of Asia, such as India, Bangladesh, and Nepal, informal waste collectors operate in extremely hazardous conditions with minimal or no access to PPE [9,10]. Studies have linked these conditions to increased prevalence of skin infections, respiratory problems, and injuries among workers [11]. Similarly, across Latin America, particularly in Brazil, Colombia, and Mexico waste pickers form a vital part of the urban recycling economy but often lack legal recognition, formal training, and adequate PPE, leaving them vulnerable to occupational hazards and social marginalization [12,13].

Although these challenges are widely known in African nations, evidence from around the world shows that this is not a unique problem. For example, informal waste workers in India report low PPE use because of cost and accessibility issues, even though they are exposed to a lot of hazardous materials and infectious waste [14]. According to a Brazilian study, despite receiving personal protective equipment (PPE), municipal waste workers do not consistently use it because of discomfort, heat, and a lack of training [15]. Even though PPE is required by law in European nations like Italy and Spain, non-compliance persists, especially among temporary waste workers and subcontracted personnel [16]. Comparably, studies conducted in the US reveal that, even in cases where safety procedures are well-defined, organizational culture, workload demands, and a lack of enforcement frequently jeopardize sanitation workers’ use of PPE [17].

Despite the importance of PPE use to minimize occupational health hazards in solid waste collection and handling processes, not much attention has been given to the predictive factors for PPE use among DWCs. Although DWCs work under unsanitary conditions in managing solid waste and are exposed to potential health-related risks, they rarely use PPE to protect themselves from occupational and health hazards [10,13,18]. There is considerable evidence to suggest that there are major barriers that contribute to the non-use of PPE among DWCs [19,20]. Some of these barriers include and are not limited to poor safety management and communication systems in waste companies in Ghana, training about safety measures, vulnerability to diseases reduced due to PPE use, poor adherence to PPE use due to fatalistic attitudes, and work exposure correlates with complacency [5,9,19,21]. These factors hamper utilization of PPE in several ways. In terms of vulnerability to diseases, DWCs frequently exposed to hazardous waste may normalize health risks, perceiving the use of PPE as unnecessary if illnesses develop gradually (e.g., chronic respiratory conditions or diseases). This false sense of adaptation reduces compliance. This is similar to Bangladeshi textile workers’ fatalism, 67% of workers thought that illnesses were “part of the job” and considered PPE to be useless [22]. With the issue of poor adherence to PPE use, when DWCs are provided PPE, that is inconsistent with rule enforcement, discomfort, or lack of perceived immediate threat leads to irregular use, undermining DWCs’ protection at their workplaces. Regarding work exposure, high-intensity workloads and time pressures may discourage proper PPE use, especially if safety protocols are seen by DWCs as slowing productivity. Collectively, these barriers reflect systemic gaps in occupational health frameworks for waste workers. This is consistent with Kenyan studies where inadequate supply chains undermined compliance [23]. Gaining an understanding of the factors that serve as barriers to the intention to use PPE helps to identify relevant targets for intervention to address structural or managerial barriers (e.g., subsidized PPE, waste companies’ managers mandates) alongside capacity building and education to promote the usage of PPE among DWCs.

Correct and consistent PPE use, as opposed to using ‘’unprotected hands” for solid waste collection and practices, has been found to prevent hazards as well as accidental health-related risks among waste workers and promote occupational safety [8,24,25]. However, evidence suggests that PPE use in Ghana remains low [9,21]. A study to determine the prevalence of PPE use during road construction activities in Ghana found that PPE use was higher for workers in foreign-owned companies compared with locally-owned companies [9]. Previous studies on PPE use among DWCs in Ghana revealed that most of them are unable to afford the cost of PPE and do not feel comfortable wearing PPE. In addition, waste companies have insufficient PPE supplies and do not provide their employees with the necessary safety provisions [9,21]. Countries like Bangladesh and Mexico have reported similar cost- and comfort-related issues, further suggesting a cross-cutting challenge [26,27]. To increase PPE, use among DWCs, it is important to gain insights into determining factors that influence the use of PPE. Previous studies conducted by [21,28] showed that PPE education and safety training result in increased PPE use, a finding that is consistent with other studies.

Several social cognitive theories and models have been applied to study how human behavior changes in relation to different health-related risks in Sub-Saharan Africa (SSA) [29–31]. Examples of such frameworks are the Theory of Planned Behavior (TPB) [32], Social Cognitive Theory [33], the Health Belief Model (HBM) [34], and the Reasoned Action Approach (RAA) [32]. The theoretical underpinnings of these theories support that an individual’s motivation (intention) to engage in a behavior is primarily determined by their evaluative assessment of the behavioral outcomes (attitude), the behavior and opinions of significant others (perceived norms), and their level of control over the behavior (perceived behavior control). Both HBM and RAA have emphasized the predictive value of perceived risk as a motivation for more healthy behavior [32,34].

Studies in China and Indonesia have similarly applied the HBM and RAA to explain PPE use in industrial and municipal sectors, with findings highlighting the critical role of perceived severity, workplace enforcement, and self-efficacy [35,36]. Previous studies in low and middle-income countries (LMIC) such as Ghana, Nigeria, Uganda, and Kenya have confirmed the role of attitudinal, normative, and control beliefs in explaining PPE use among DWCs [12,18,37,38]. However, in Ghana, the evidence is limited to qualitative studies that do not allow for a quantification of the relevance of the different determinants in predicting precautionary motivation with regard to PPE use. Our study addresses a crucial gap by investigating DWCs in the Ho Municipality, Ghana, a population characterized by unique socio-economic vulnerabilities, informal work arrangements, and distinct cultural contexts that significantly influence health and safety behaviors [7,8].

Critically, previous studies have examined either the HBM or the RAA in isolation and have rarely incorporated organisational rule-enforcement. The present study is the first to integrate HBM and RAA constructs with perceived managerial enforcement in a single partial least squares structural equation model, thereby quantifying the separate and joint contributions of individual cognition and organisational climate to PPE-use intentions. Using an empirical and theory-based cross-sectional survey tool, this study examined the socio-cognitive determinants of PPE use among DWCs in an urban Ghanaian town. An understanding of the social and cognitive determinants for PPE use can inform the design of intervention programs to promote PPE use in the study setting and across similar environments in Ghana and beyond.

## Materials and methods

### Study design

A quantitative cross-sectional study design [14] was conducted to assess the socio-cognitive determinants of PPE use among DWCs (n = 344) in the Ho Municipality of Ghana. A cross-sectional survey was deemed appropriate given that data would be collected at a single time point without any follow-up. Data were collected via structured face-to-face administered questionnaires to assess PPE utilization patterns and associated risk factors.

### Study setting and population

The research was carried out in the Ho Municipality of the Volta Region of Ghana. The Volta Region currently has 18 administrative districts, with the Ho Municipality serving as the administrative capital. The projected 2018 population of the Ho Municipality was 213,960 at the time of data collection, with 51 percent of the population being female [15]. The municipality is known as the commercial hub of the Volta Region due to its strategic location and proximity to the Republic of Togo, as well as easy access to district and regional capitals such as Accra. The municipality was chosen for the study because of its solid waste management issues and the state of the municipality’s environment, both of which need to be addressed significantly. Data were collected from the two waste management companies in the Ho Municipality (see reference [37] for more information on the waste management companies).

The study was conducted among DWCs in Ho Municipality of Ghana who worked for two solid waste companies. The waste management companies and respondents were kept anonymous (the two participating companies are known as Company A and B). Waste collection, waste processing, waste transportation, and waste disposal in designated dumping sites are all part of DWCs’ daily work schedules. The two solid waste companies analyzed in this study employ DWCs with notably low educational attainment and wages compared to other sectors in the region [25], a characteristic that may influence occupational health behaviors and risk exposure. The estimated study population consisted of 1,025 DWCs.

### Inclusion and exclusion criteria

The specific inclusion criteria were that DWCs must work for at least one year and above to be eligible to take part in the study. DWCs who did not provide written consent and were ill or absent at the time of data collection were excluded from the study. In addition, respondents were restricted to adult DWCs aged ≥18 years, as Ghana’s labor laws prohibit formal employment in hazardous waste handling for minors. No respondents under 18 were enrolled, aligning with international ethical guidelines for occupational health research. Thus, workers less than 18 years, on medical/annual leave, or declining written consent were excluded.

### Study instruments

The construction of the questionnaire was informed by a review of the literature on the determinants of taking protective measures by DWCs, previous qualitative research by the team of authors in the Ho municipality on health risks and preventive measures among DWCs and their managers and supervisors [25,37], and the theoretical framework of the HBM, and the RAA. The study utilized a structured questionnaire which was divided into seven (7) sections. The first section of the questionnaire included socio-demographic information such as gender, age, marital status, ethnicity, educational level, job roles, and the number of years at the workplace. These items were placed at the beginning of interviews on purpose to foster understanding between the research team and the study participants. The subsequent sections measured constructs of the HBM and RAA that used five-point Likert scales (1 = strongly disagree; 5 = strongly agree). These included measures of perceived susceptibility to occupational-related health problems; perceived severity of occupational-related health problems; perceived benefits of using PPE; perceived barriers to use PPE; identified cues to action; perceived self-efficacy of using PPE; attitude toward PPE use; subjective norms towards PPE use; behavioral beliefs (i.e., normative beliefs are individuals’ beliefs about the extent to which other people who are important to them think they should, or should not perform particular behaviors) towards PPE use, and intention to use PPE (for the full questionnaire, see S1 Appendix). Intention was the principal outcome variable; socio‑cognitive constructs served as independent variables.

### Quality control measures

To ensure construct and face validity, the questionnaires were developed in English, reviewed by a panel of 5 experts (occupational health specialists, and waste management professionals) to assess relevance and clarity using a 4-point Likert scale (CVI = 0.82), translated into Ewe, then translated back into English. Discrepancies between the two first and second English versions of the questionnaire were resolved through discussion among the research team. Two (2) research assistants and two (2) interpreters who are natives of the study setting with prior knowledge of waste management practices were trained to assist in all field data collection, standardized question delivery, ethical protocols (e.g., Informed consent) data recording, and also to minimize interviewer bias, in addition to mock interviews with feedback. A pilot study was done among 50 participants with similar socioeconomic backgrounds to the respondents of the study population to test for validity and reliability from 01/05/2018 to 15/05/2018. This was done using the research instrument developed for the study to assess how easy it would be for respondents to understand the questions. Except for minor textual changes, no content changes were made to the instrument.

The data for this study were collected through researcher-administered, face-to-face interviews using structured questionnaires. This approach was chosen to ensure clarity and completeness for trained research assistants to verbally explain questions to DWCs, address ambiguities in real-time, and minimize missing responses. Also, to account for literacy variability given potential variations in literacy levels among DWCs, verbal administration improved accessibility and data quality. In addition, to enhance contextual validity to allow face-to-face interactions with the researchers to observe non-verbal cues and cross-check responses (e.g., verifying self-reported PPE use with visual inspections where possible). However, the rationale for the research team not using Self-Administered/Online Methods were due to low feasibility with regards to limited smartphone/internet access among the target population (DWCs) made tools like Google Forms impractical. Moreover, the issue of research team engagement in face-to-face interactions with DWCs fostered trust and higher participation rates compared to self-administered methods.

### Sampling and data collection procedure

The study’s field survey was from May to July 2018 covering typical working hours for DWCs in the Ho Municipality (e.g., 5:00 am to 10:00 am), coinciding with their active waste collection schedules. This timing ensured that DWCs were available and engaged in their routine tasks, enhancing the ecological validity of responses regarding PPE use. The survey instruments (e.g., questionnaires, and observational checklists) were administered on-site during short breaks or upon completion of shifts to minimize disruption in the work. Each interview/questionnaire session lasted approximately 10–15 minutes, while direct observations spanned 2–3 hours per work session to assess real-time PPE compliance. Adjustments were made for DWCs who opted to participate before or after shifts to accommodate their preferences.

A multi-stage sampling procedure was used to select the sample [16]. In the first stage, we divided the Ho Municipality into five (5) zones purposely after visiting the area and consulting with the local Municipal Assembly and regulatory bodies such as Environmental Health inspectors. In the second stage, 20 sub-zones (four from each Zone) were chosen at random, and finally, DWCs were chosen at random from a list submitted by solid waste company managers and supervisors [37]. The use of a multi-stage sampling procedure allows practically for primary data collection for large study respondents that are geographically dispersed, reduces the costs and time associated with data collection, and provides flexibility, for the research team to break down the participants as often as necessary to create the sample population needed.

Using Cochran’s single-population proportion formula, the preliminary requirement was 384 respondents [39]. Applying the finite-population correction for N = 1,025 produced n = 278. To offset an anticipated 30% non-response, the target was inflated to 400 (278/ 0.70). Ultimately, 344 fully completed questionnaires were returned, yielding an 86% response rate; only these complete cases were included in the analyses.

### Data analyses

SPSS version 25 and SmartPLS 3.3.9 [40] were used for the data analysis. Only fully completed questionnaires (n = 344) were retained for analysis. In the analysis, descriptive statistics of the study respondents such as socio-demographic characteristics were generated into percentages and frequencies as demonstrated in Table 1. Partial least squares structural equation modeling (PLS-SEM) procedure, which has the predictive ability to evaluate several regression models or equations concurrently [26], was then used to examine the study model and its paths [27]. Thus, the measurement model including the reliability and validity of measures was examined using the PLS algorithm with default settings. Internal consistency was satisfactory: Cronbach’s α ranged from 0.673–0.906, composite reliability from 0.815 – to 0.934, and average variance extracted (AVE) from 0.511 to 0.779. Next, the structural model was evaluated using the bootstrapping technique with 5000 resamples, and finally the blindfolding procedure [17,27]. The figure of 5,000 resamples was adopted in line with Hair et al.’s [27] guidance that at least 5,000 subsamples are necessary for stable estimates and resamples were generated by random sampling with replacement within SmartPLS [40]. Before bootstrapping, univariate normality was evaluated using the Shapiro–Wilk test; most indicators departed from normality (p < .05). This justified the use of PLS-SEM, which does not assume multivariate normality.

**Table 1:** Demographics characteristics of respondents(n = 344).

Variables	Frequency (344)	Percent (%)
***Age***		
**Mean, SD (22.18, ± 11.09)**		
<20 years	157	45.6
20-29 years	90	26.2
30-39 years	69	20.1
≥40 years	28	8.1
***Sex***		
Male	175	50.9
Female	169	49.1
***Ethnicity***		
Akan	30	8.7
Ewe	292	84.9
Ga	19	5.5
Hausa	3	0.9
***Religion***		
Christianity	293	85.2
Islam	18	5.2
Traditional	33	9.6
***Marital Status***		
Single	73	21.4
Married	204	59.8
Co-habiting	9	2.7
Divorced	17	5.0
*Widowed*	38	11.1
***Educational Level***		
No education	42	12.3
MSLC	68	19.8
Junior High School	125	36.5
Vocational training	44	12.9
Senior High School	18	5.3
Tertiary	45	13.2
***Job role***		
DWCs (Cleaner, Sweeper, Driver)	283	83.2
Manager	24	7.1
Supervisor	33	9.7
***Number of years at the workplace***		
1-5 years	167	48.8
6-10 years	62	18.1
11-15 years	6	1.8
16-20 years	98	28.6
Over 20 years	9	2.6
***Company Category***		
Category A	329	95.6
Category B	15	4.4

*Note: SD = standard deviation; n = sample size; DWCs = Domestic Waste Collectors; MSLC = Middle School Leaving Certificate*

### Ethical approval

The study was approved by The Ethical Review Committee Psychology and Neuroscience (ERCPN) of the Faculty of Psychology and Neuroscience, Maastricht University (approved research line ERCPN-188_10_02_2018), and the Ghana Health Service Ethics Review Committee (GHSERC 08/05/17). Permission was also obtained from the waste management companies from which the respondents were recruited. The first page of the questionnaire provided information about the project, the survey, and the voluntary nature of their participation. The description outlined the project’s goal and the risks associated with not wearing PPE while emphasizing the anonymity and confidentiality of participants. In addition, trained research assistants explained the project and the survey to participants, who were informed that they could withdraw from the study at any moment without penalty. The respondents provided written informed consent before participating in the study. Additional information regarding the ethical, cultural, and scientific considerations specific to inclusivity in global research is included in the S2 Appendix.

## Results

### Socio-demographic profile of the respondents

The final sample consisted of 344 DWCs. Close to half of the participants (n = 157, 45.6%) were aged < 20 y (M = 22.18, SD = 11.09; range = 18–64 years). Most participants were male (n = 175, 50.9%), identified as Ewe (n = 292, 84.9%), professed Christianity (n = 293, 85.2%), and were married (n = 204, 59.8%).

Furthermore, most respondents were DWCs (83.2%; n = 175) and achieved a junior high school education (n = 125, 36.5%). Lastly, most had 1–5 years of experience (n = 167, 48.8%), and belonged to Company A (n = 329, 95.6%).

### Measurement Model Assessment (Reliability and Validity Results)

This section reports the reliability and validity of the construct measures as advocated by [27]. The item loadings, VIF, construct reliability coefficients, and convergent validity (AVE) reported in Table 2 prove that all construct indicators have significant factor loadings (p < 0.001) with no multicollinearity issues (VIF < 5), and all the latent variables exhibit adequate reliability and convergent validity as the values of composite reliability (CR), and the average variance extracted (AVE) for each latent variable are greater than the minimum threshold limit of 0.70 and 0.50, respectively. Thus, there is acceptable reliability and convergent validity of the constructs [27,35,36]. Further, to check the discriminant validity of the constructs, the Heterotrait-Monotrait ratio (HTMT) criterion was used as reported in Table 3. The HTMT values are less than 0.90 as recommended [41,42], thus, confirming the presence of constructs’ discriminant validity.

**Table 2:** Construct reliability and convergent validity results.

Indicator	Loadings	*SE*	*t*-statistics	*P < .001*	VIF	CA	rho_A	CR	AVE
***Attitude (Att)***									
Att1	0.864	0.024	35.331	0.000	3.219	0.845	0.863	0.905	0.761
Att2	0.908	0.016	56.793	0.000	3.638				
Att3	0.843	0.022	38.222	0.000	1.543				
***Perceived Barriers/Behavioral Control (Bar)***									
Bar1	0.648	0.071	9.130	0.000	2.226	0.877	0.935	0.892	0.511
Bar2	0.749	0.050	14.901	0.000	2.783				
Bar3	0.668	0.072	9.265	0.000	3.217				
Bar4	0.618	0.062	9.988	0.000	1.681				
Bar5	0.599	0.076	7.853	0.000	2.039				
Bar6	0.840	0.027	31.657	0.000	2.525				
Bar7	0.761	0.035	21.689	0.000	1.742				
Bar8	0.795	0.037	21.727	0.000	2.316				
***Behavioral beliefs (Bel)***									
Bel1	0.828	0.038	21.916	0.000	1.243	0.673	0.713	0.815	0.596
Bel3	0.690	0.062	11.139	0.000	1.327				
Bel4	0.790	0.038	20.853	0.000	1.441				
***Perceived Benefits (Ben)***									
Ben1	0.812	0.035	22.931	0.000	2.006	0.827	0.836	0.879	0.593
Ben2	0.840	0.028	30.532	0.000	2.393				
Ben3	0.762	0.040	18.962	0.000	1.895				
Ben4	0.740	0.048	15.380	0.000	1.765				
Ben5	0.689	0.056	12.197	0.000	1.630				
***Cues to Action (Cues)***									
Cues1	0.707	0.038	18.726	0.000	1.597	0.864	0.867	0.894	0.513
Cues2	0.692	0.043	16.215	0.000	3.799				
Cues3	0.699	0.045	15.373	0.000	4.025				
Cues4	0.769	0.034	22.936	0.000	2.147				
Cues5	0.684	0.046	14.844	0.000	1.783				
Cues6	0.691	0.046	14.865	0.000	1.678				
Cues7	0.765	0.039	19.729	0.000	2.173				
Cues8	0.717	0.041	17.458	0.000	1.778				
***Behavioral Intention (Int)***									
Int1	0.715	0.041	17.239	0.000	1.570	0.869	0.875	0.905	0.658
Int2	0.792	0.035	22.384	0.000	2.132				
Int3	0.867	0.025	34.559	0.000	2.692				
Int4	0.855	0.023	37.909	0.000	2.479				
Int5	0.817	0.025	32.947	0.000	2.215				
***Management enforcing PPE use (Mgt)***									
Mgt1	0.854	0.023	36.423	0.000	2.704	0.875	0.881	0.906	0.618
Mgt2	0.821	0.033	25.129	0.000	2.521				
Mgt3	0.811	0.034	23.902	0.000	2.231				
Mgt4	0.766	0.037	20.784	0.000	1.860				
Mgt5	0.731	0.044	16.681	0.000	1.936				
Mgt6	0.724	0.051	14.270	0.000	1.933				
***Self-Efficacy (SEffic)***									
SEffic1	0.644	0.060	10.766	0.000	1.732	0.836	0.852	0.880	0.552
SEffic2	0.724	0.043	16.684	0.000	1.659				
SEffic3	0.826	0.028	29.987	0.000	2.444				
SEffic4	0.839	0.020	41.097	0.000	2.446				
SEffic5	0.763	0.037	20.802	0.000	1.926				
SEffic6	0.636	0.050	12.612	0.000	1.343				
***Subjective Norms (Snorms)***									
SNorm1	0.825	0.031	26.845	0.000	1.559	0.786	0.823	0.856	0.599
SNorm2	0.811	0.032	25.049	0.000	1.601				
SNorm3	0.789	0.047	16.733	0.000	2.074				
SNorm4	0.662	0.069	9.620	0.000	1.699				
***Perceived Susceptibility (Scep)***									
Scep1	0.882	0.021	41.456	0.000	3.198	0.906	0.911	0.934	0.779
Scep2	0.897	0.016	56.827	0.000	3.052				
Scep3	0.903	0.017	52.258	0.000	3.669				
Scep4	0.847	0.028	30.273	0.000	2.631				
***Perceived Severity (Sev)***									
Sev1	0.773	0.031	24.951	0.000	1.939	0.879	0.895	0.906	0.581
Sev2	0.819	0.025	33.261	0.000	2.446				
Sev3	0.789	0.032	24.526	0.000	2.310				
Sev4	0.592	0.062	9.622	0.000	1.427				
Sev5	0.767	0.027	28.463	0.000	1.806				
Sev6	0.811	0.029	28.105	0.000	2.450				
Sev7	0.759	0.040	19.051	0.000	2.199				

**Table 3:** Discriminant validity using Heterotrait-Monotrait Ratio of Correlations (HTMT) Criterion.

Constructs	1	2	3	4	5	6	7	8	9	10	11
1. Att	–										
2. Bar	0.208	–									
3. Bel	0.634	0.394	–								
4. Ben	0.532	0.383	0.701	–							
5. Cues	0.484	0.369	0.609	0.709	–						
6. Int	0.626	0.421	0.647	0.777	0.850	–					
7. Mgt	0.384	0.172	0.419	0.484	0.485	0.541	–				
8. SEffic	0.616	0.351	0.572	0.559	0.633	0.719	0.428	–			
9. SNorm	0.539	0.213	0.426	0.602	0.404	0.479	0.481	0.384	–		
10. Scep	0.326	0.266	0.296	0.374	0.330	0.464	0.312	0.299	0.359		
11. Sev	0.441	0.256	0.303	0.315	0.227	0.322	0.317	0.337	0.486	0.592	–

The results in Table 4 demonstrate that all the predictors have significant positive correlations with intention with r = .10−.23 signifying a small effect, r = .24−.36 demonstrating a moderate effect, and r ≥ .37 signifying a large effect [43]. Specifically, we found strong positive univariate associations with the intention for attitude, susceptibility, behavioral beliefs, subjective norm, perceived benefits, cues to action, self-efficacy, and management. Severity and perceived barriers had moderate positive associations with intentions.

**Table 4:** Descriptive statistics and correlational matrix.

Variables	M	SD	1	2	3	4	5	6	7	8	9	10	11
1. Int	3.85	0.817	1										
2. Att	3.86	0.850	.533^**^	1									
3. Scep	3.28	1.069	.408^**^	.279^**^	1								
4. Sev	3.24	0.891	.281^**^	.379^**^	.526^**^	1							
5. Bel	3.43	0.795	.520^**^	.412^**^	.258^**^	.233^**^	1						
6. SNorm	3.53	0.869	.392^**^	.436^**^	.296^**^	.403^**^	.256^**^	1					
7. Ben	3.90	0.722	.654^**^	.441^**^	.319^**^	.265^**^	.485^**^	.489^**^	1				
8. Bar	3.26	0.885	.363^**^	.160^**^	.240^**^	.223^**^	.346^**^	.150^**^	.320^**^	1			
9. Cues	3.83	0.703	.757^**^	.422^**^	.312^**^	.207^**^	.471^**^	.369^**^	.605^**^	.340^**^	1		
10. SEffic	3.88	0.715	.624^**^	.516^**^	.261^**^	.294^**^	.440^**^	.315^**^	.462^**^	.294^**^	.563^**^	1	
11. Mgt	3.73	0.947	.531^**^	.376^**^	.334^**^	.337^**^	.350^**^	.461^**^	.452^**^	.158^*^	.508^**^	.442^**^	1

**. Correlation is significant at the 0.01 level (2-tailed).

*. Correlation is significant at the 0.05 level (2-tailed).

### Predictors of intention to use PPE (Structural model assessment)

**Fig 1:**
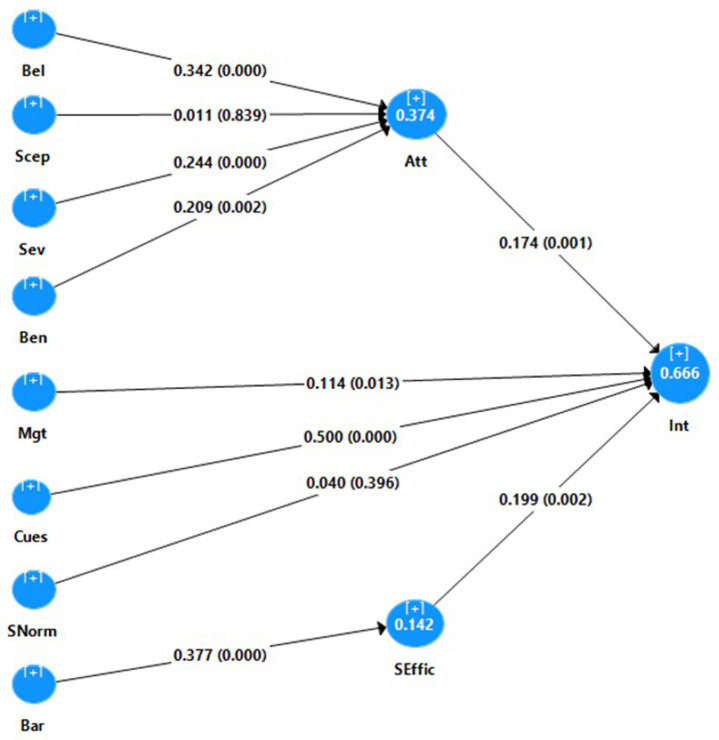
Structural model of factors that affect the use of PPE.

Succeeding the fulfillment of the reliability and validity of the measurement model, the structural model was examined including the structural relationships, model’s predictive power, and relevance [27,44]. As revealed in the model’s summary section of Table 5 and Fig 1, the beliefs, susceptibility, severity and perceived benefit explained 37.4% of the variance in attitude (Q^2 ^= 0.261), and attitude, cues to action, management, self-efficacy, subjective norm accounted for 66.6% of the variation in intention (Q^2 ^= 0.424). Lastly, perceived barriers contribute to 14.2% of the variance in self-efficacy (Q^2 ^= 0.074). Thus, the structural model exhibits good predictive power, accuracy, and relevance [27,45–47].

**Table 5:** Structural model results.

Paths	*B*	*SE*	*t-*statistics	*p*-values	BCa Confidence Interval	
					LL	UL
					2.50%	97.50%
***Direct Effects***						
Bel - > Att	0.342	0.058	5.952	0.000	0.224	0.450
Scep - > Att	0.011	0.056	0.203	0.839	−0.103	0.121
Sev - > Att	0.244	0.051	4.804	0.000	0.141	0.340
Ben - > Att	0.209	0.068	3.066	0.002	0.074	0.340
Att - > Int	0.174	0.053	3.285	0.001	0.070	0.278
Cues - > Int	0.500	0.054	9.190	0.000	0.392	0.602
Mgt - > Int	0.114	0.046	2.480	0.013	0.028	0.212
SEffic - > Int	0.199	0.065	3.081	0.002	0.076	0.325
SNorm - > Int	0.040	0.047	0.849	0.396	−0.052	0.131
Bar - > Seffic	0.377	0.045	8.413	0.000	0.264	0.449
***Indirect Effects (Mediating Effects)***						
Ben - > Att - > Int	0.036	0.020	1.848	0.065	0.009	0.087
Bel - > Att - > Int	0.060	0.019	3.073	0.002	0.026	0.104
Scep - > Att - > Int	0.002	0.010	0.197	0.844	−0.019	0.023
Sev - > Att - > Int	0.042	0.015	2.875	0.004	0.018	0.077
Bar - > SEffic - > Int	0.075	0.029	2.594	0.010	0.023	0.131
***Model’s summary***				**R** ^**2**^	**R**^**2**^ **Adjusted**	**Q** ^**2**^
Att				0.374	0.367	0.261
Int				0.666	0.661	0.424
SEffic				0.142	0.140	0.074

Furthermore, the complete bootstrapping procedure using 5,000 resamples with no significant changes revealed that beliefs (*β* = 0.342; *p* = 0.000; BCa 95% CI [0.224; 0.450]), severity (*β* = 0.244; *p* = 0.000; BCa 95% CI [0.141; 0.340]), and perceived benefits (*β* = 0.209; *p* = 0.002; BCa 95%CI [0.074; 0.340]) had significant positive direct effects on attitude. However, susceptibility (*p* = 0.839) failed to have significant direct influence on attitude.

Regarding the predictors of intention, intention was significantly positively and directly influenced by attitude (*β* = 0.174; *p* = 0.001; BCa 95%CI [0.070; 0.278]), cues to action (*β* = 0.500; *p* = 0.000; BCa 95%CI [0.392; 0.602]), management (*β* = 0.114; *p* = 0.013; BCa 95%CI [0.028; 0.212]), and self-efficacy (*β* = 0.199; *p* = 0.002; BCa 95%CI [0.076; 0.325]). But, the effect of subjective norm on intention was not significant (*p* = 0.396). Lastly, as expected, the barriers perceived had a significant positive direct effect on self-efficacy (*β* = 0.377; *p* = 0.000; BCa 95%CI [0.264; 0.449]).

Further examination of the paths in Table 5 revealed three significant indirect effects. Specifically, beliefs (*β* = 0.060; *p* = 0.002; BCa 95% CI [0.026; 0.104]), severity (*β* = 0.042; *p* = 0.004; BCa 95% CI [0.018; 0.077]) and perceived benefit (β = 0.036; p = 0.065; BCa 95% CI [0.009; 0.087]) had significant indirect effects on intention through attitude. Thus, attitude mediated the influence of beliefs, severity, and perceived benefit on intention. Similarly, self-efficacy mediated the effect of perceived barriers on intention (*β* = 0.075; *p* = 0.010; BCa 95%CI [0.023; 0.131]).

## Discussion

This study used an integrated framework based on the HBM and RAA to identify socio-cognitive factors associated with DWCs’ PPE use intentions. Overall, the model indicated that DWCs’ intention to use PPE was driven primarily by their own attitudes, confidence, and cues/reminders, rather than social approval. Specifically, a more positive attitude toward PPE, stronger cues to action (including perceived management enforcement of PPE rules), and higher self-efficacy each showed significant positive associations with the intention to use PPE. By contrast, the direct influence of subjective norms on intention was not significant. We also found that DWCs’ attitudes were shaped by their risk perceptions and beliefs about PPE: those perceiving greater severity of work-related hazards, recognizing more benefits of PPE use, and holding positive behavioral beliefs had more favorable attitudes toward PPE. Additionally, perceived barriers to PPE use were inversely related to self-efficacy (i.e., workers who saw more obstacles felt less confident about using PPE). Together, these socio-cognitive factors accounted for a substantial portion of the variance in PPE use intention (about 67% of the total variance), underscoring the strong explanatory power of the combined HBM–RAA model. Indirect pathway analysis further supported the theoretical framework: for instance, attitude mediated the effects of underlying beliefs, perceived severity, and perceived benefits on intention, while self-efficacy mediated the effect of perceived control on intention.

Our findings are broadly consistent with prior research on PPE use in various occupational settings [48,49]. For example, a study among industrial workers in Ethiopia reported that higher perceived susceptibility and severity of health risks, greater self-efficacy, and fewer perceived barriers were all significant predictors of good PPE utilization [50]. This alignment suggests that when workers understand the likelihood and seriousness of workplace hazards and feel capable of protecting themselves, they are more inclined to adopt PPE – a pattern evident among both our DWCs and the Ethiopian factory workers. Similarly, studies in other contexts have observed the importance of these socio-cognitive factors. Among farmers handling pesticides, key HBM constructs such as perceived risk severity, strong cues to action, and the perceived benefits of PPE were found to positively influence intentions to use PPE. These parallels imply that workers’ awareness of hazard consequences and their belief in the efficacy of PPE are universally critical for motivating protective behavior. Notably, the shared importance of risk perception and self-efficacy across different regions might reflect common underlying challenges in workplace safety education and awareness. In our context, this may be due to similar workplace conditions and levels of hazard awareness, as well as comparable educational backgrounds among workers. Despite the overall agreement with prior studies, some of our results deviated from expectations and highlight context-specific nuances. Most prominently, perceived social norms did not significantly affect DWCs’ PPE intentions in our study, whereas research from other regions (e.g., the Middle East and East Asia) has found that social pressure and normative beliefs can play a role in PPE use. One possible explanation is that DWCs rely more on personal risk-benefit evaluations and official rules than on peer opinions when deciding to use PPE. It could also be that our measure of subjective norm (workers’ perception of whether important others think they should use PPE) did not capture the informal influences present on the job. For instance, direct encouragement or modeling by co-workers might influence PPE use in practice, even if workers do not explicitly recognize peer approval as a factor. Another noteworthy finding was that perceived susceptibility (the perceived likelihood of being harmed by workplace hazards) was not a strong standalone predictor in our model. This contrasts with the Ethiopian study, in which higher susceptibility was associated with better PPE use [50]. It is possible that in our sample, DWCs generally already feel susceptible to injury or illness, reducing the variability of this perception, or that they consider the severity of potential outcomes more important than abstract probability. These unexpected results underscore the importance of context – factors like workplace culture, enforcement practices, and group dynamics may moderate how socio-cognitive determinants translate into safety intentions.

### Theoretical implications

Our findings underscore the value of integrating the HBM and RAA frameworks – along with organizational factors to advance the theoretical understanding of PPE use behavior. The combined model demonstrated that both HBM constructs (e.g., perceived severity, benefits, barriers, cues to action) and RAA constructs (e.g., attitude, self-efficacy as a proxy for perceived behavioral control, subjective norm) jointly explain a substantial proportion of variance (67%) in DWCs’ intention to use PPE. This high explanatory power suggests that a multi-theory approach can capture a wider range of socio-cognitive influences on protective behavior than any single theory alone. For instance, risk perception variables from HBM (such as the perceived likelihood and seriousness of injury) were found to shape workers’ attitudes toward PPE, while behavioral belief-based attitude and self-efficacy from RAA directly drove intention. This indicates that both the perception of risk and the evaluation of PPE’s benefits/feasibility are critical motivators. Notably, our study showed that perceived management rule enforcement, an external contextual factor, had a significant positive effect on PPE use intention (comparable in magnitude to personal attitudinal factors). The inclusion of this managerial enforcement element – conceptually akin to a strong cue to action or an environmental facilitator – extends traditional behavior models by acknowledging that institutional safety rules and their enforcement can directly motivate worker compliance. This theoretical extension is particularly important in occupational settings where formal regulations and supervision play a role in shaping individual behavior, adding a new dimension to HBM and RAA in the context of workplace safety.

Comparing our results with prior studies further highlights the theoretical contributions. In line with our findings, a study of factory workers in Ethiopia reported that higher perceived susceptibility and severity of hazards, greater self-efficacy, and fewer perceived barriers were associated with better PPE usage. Similarly, research using HBM in an agricultural context (Iran) and safety behavior studies in China have observed that internal drives (like risk awareness and confidence) and external triggers can jointly influence protective behavior. These parallels across diverse contexts suggest that our integrative socio-cognitive model has broad applicability in occupational health theory, reinforcing the generalizability of HBM/RAA constructs for PPE compliance. However, our results also revealed a nuanced divergence: subjective norms did not significantly predict PPE intention in our sample, whereas some studies in Asia did find social normative influences to be important. This discrepancy implies that the role of peer influence may depend on context – for Ghanaian waste collectors, coworkers’ opinions or example might be less salient than personal conviction and management directives. From a theoretical standpoint, this finding invites refinement of the RAA/TPB normative component for similar settings, perhaps by distinguishing between injunctive norms (what others think one should do) and descriptive norms (what others actually do) or considering the effect of direct peer pressure. In sum, the integration of HBM and RAA, enriched with an organizational enforcement factor, advances our theoretical understanding by providing a more comprehensive model of PPE-use behavior. It demonstrates the importance of multi-level determinants – individual cognitive factors and institutional cues – and sets the stage for future occupational safety research to adopt and further test such combined theoretical frameworks in explaining and predicting protective behaviors in various work environments.

### Practical implications

The study’s findings translate into actionable insights for public health officials, waste-management companies, and safety training providers aiming to improve PPE use among DWCs. First, education and training interventions should be prioritized to address the identified socio-cognitive gaps. Because positive attitude toward PPE was a strong predictor of intent, and attitude in turn was shaped by risk perceptions and beliefs about benefits, training programs must emphasize hazard awareness and the tangible benefits of consistent PPE use. Public health and safety trainers can develop targeted modules that vividly communicate the severity and susceptibility of work-related injuries (for example, sharing local case studies of injuries prevented by proper PPE) to increase risk perception and personal relevance. Such trainings should also highlight the protective advantages of gloves, masks, boots, and other gear in preventing disease or injury, thereby fostering more positive outcome expectations. Indeed, previous studies in Ghana and other low-resource settings have shown that tailored PPE education and safety training significantly improve workers’ PPE usage rates. These educational efforts will not only improve knowledge but can serve as continuous cues to action reinforcing the habit of PPE use through reminders, demonstrations, and visual signage at the workplace.

Second, the role of management is crucial: waste-management companies should strengthen enforcement of PPE policies and ensure supportive working conditions. Our findings that perceived rule enforcement by management is a significant motivator for PPE use indicate that stricter supervision and clear organizational safety rules can drive behavior change. Company managers and supervisors need to consistently enforce PPE requirements – for instance, by mandating PPE at the start of each shift, conducting spot checks, and instituting accountability measures for non-compliance. Importantly, enforcement should be paired with facilitation: firms must provide adequate PPE supplies (of the right sizes and comfort) to all workers at no cost. Prior research in Ghana revealed that many waste workers lack or avoid PPE due to cost, discomfort, and insufficient supply from employers. Addressing these barriers through budget allocation for PPE procurement and replacement will improve workers’ self-efficacy and willingness to use the equipment. Additionally, establishing a safety culture where supervisors lead by example (wearing PPE themselves and encouraging peer support for compliance) could compensate for the weaker influence of subjective norms in this context. Public health authorities and labor regulators also have a part to play by setting and enforcing industry-wide standards – for example, integrating waste sector PPE compliance into municipal contracts or labor inspections. Clear policy guidelines (such as municipal bylaws or company-level Standard Operating Procedures) that mandate PPE use, coupled with training on their importance, will create an environment where wearing protective gear is the default expectation.

Finally, these implications extend to other low-resource occupational settings beyond urban waste collection. In many developing regions, workers face similar challenges of limited resources, low risk awareness, and lax enforcement of safety rules. The approaches indicated by our study – enhanced hazard communication, empowerment of workers through training, and diligent managerial enforcement of safety protocols – are broadly applicable to improve occupational health outcomes where PPE compliance is suboptimal. Interventions built on the socio-cognitive determinants (like shaping attitudes and boosting self-efficacy) can often be implemented with relatively low cost but high impact, making them ideal for resource-constrained settings. By combining behavior-change education with organizational policy action, stakeholders can significantly raise PPE usage and protect workers’ health even in the face of economic limitations. In short, public health officials, company managers, and safety trainers should collaborate to translate these findings into practice: develop comprehensive PPE programs that integrate training, consistent rule enforcement, and ongoing risk communication. Such multi-faceted efforts, informed by our study’s evidence, will improve safety behavior among DWCs and can be adapted to enhance occupational safety across similar high-risk industries.

### Strengths and limitations

This research contributes to the literature by examining PPE use determinants in an understudied population (DWCs in Ghana) using a comprehensive theoretical approach. A key strength of our study is the integration of constructs from both the HBM and RAA, which allowed us to capture a wide range of influences on PPE use intention. This integrated model explained a high proportion of variance in intention, suggesting it can offer richer explanatory power than single-theory models. However, the use of an extensive combined framework also introduced certain challenges. The survey instrument was quite lengthy (107 items in total), raising the potential for respondent fatigue or reduced data quality. Future studies might streamline the measurement of similar constructs to minimize burden. In addition, our cross-sectional study design limits causal inference – we can identify associations but cannot establish the direction of cause and effect between socio-cognitive factors and PPE use intentions. Longitudinal studies or interventions would be useful to determine how changes in these determinants lead to changes in PPE use behavior over time. All data were self-reported, so responses may be subject to biases such as social desirability or recall bias. This means that some DWCs might have overestimated their intentions or provided socially acceptable answers regarding safety practices. Another limitation is the generalizability of our findings. The study sample was drawn from a single municipality, and the majority of participants shared similar backgrounds within that region. As a result, the results may not fully represent DWCs in other parts of Ghana or in different countries. Finally, while our integrated theoretical model proved useful, many of the significant predictors aligned with well-established HBM components (e.g., risk perceptions, perceived benefits, self-efficacy). This overlap suggests that the added value of incorporating all RAA elements (such as subjective norms) should be interpreted with caution. In contexts like ours where peer norms showed little effect, a more parsimonious model focusing on threat appraisal and efficacy beliefs might perform nearly as well. Nevertheless, using the combined approach provided a holistic view and helped confirm that multiple socio-cognitive pathways – from individual threat appraisal to organizational cues – jointly influence DWCs’ motivation to use PPE.

## Conclusion

This cross-sectional study identified several key socio-cognitive determinants of PPE use intention among DWCs in Ghana, including workers’ attitudes toward PPE, self-efficacy, cues to action (such as reminders or prompts), and the perceived enforcement of safety rules by management. Together, these factors – drawn from the integrated HBM and RAA theoretical framework explained a large proportion of the variance in PPE-use intention, highlighting the dominant influence of both personal beliefs and organizational context on safety behavior. Our findings contribute to the body of knowledge by quantitatively validating the roles of these determinants in a low-resource, sub-Saharan African setting, thereby expanding the application of health behavior theory to the domain of occupational safety. This work addresses a gap in the literature (hitherto dominated by qualitative insights in Ghana) by providing empirical evidence on which specific belief constructs most strongly drive PPE compliance. The study’s integrative approach demonstrates the added value of combining classic health behavior models for understanding PPE use, and the insights gained can inform the design of theory-based interventions and policies tailored to waste workers’ needs. Future research should build on this foundation by employing longitudinal and intervention-based study designs. Long-term cohort studies or experimental trials are recommended to establish causal relationships between the identified socio-cognitive factors and actual PPE usage, and to test the effectiveness of targeted intervention programs (e.g., training workshops, policy enforcement strategies) in improving PPE adherence. Such future studies will not only verify the causal influence of socio-cognitive determinants on PPE behavior but also help translate our findings into sustained, real-world improvements in occupational health and safety.

## Supporting information

S1 Appendix Survey instrument for domestic waste collectors. (DOC)

S2 Appendix Inclusivity in global research. (DOC)

## References

[1] Lasota AM, Hankiewicz K. Self-reported fatigue and health complaints of refuse collectors. Cent Eur J Oper Res. 2019;28(2):633–43. doi: 10.1007/s10100-019-00637-w

[2] Abdalla S, Apramian SS, Cantley LF, Cullen MR. Occupation and Risk for Injuries. In: Disease Control Priorities, Third Edition (Volume 7): Injury Prevention and Environmental Health. The World Bank; 2017. p. 97–132.

[3] Baral YR. Waste Workers and Occupational Health Risks. Int J Occup Saf Heal. 2018;8:1.

[4] van Kampen V, Hoffmeyer F, Seifert C, Brüning T, Bünger J. Occupational health hazards of street cleaners - a literature review considering prevention practices at the workplace. Int J Occup Med Environ Health. 2020;33(6):701–32. doi: 10.13075/ijomeh.1896.01576 32939096

[5] Jafaralilou H, Zareban I, Hajaghazadeh M, Matin H, Didarloo A. The impact of theory-based educational intervention on improving helmet use behavior among workers of cement factory, Iran. J Egypt Public Health Assoc. 2019;94(1):1. doi: 10.1186/s42506-018-0001-6 30679877 PMC6323090

[6] Amabye TG. Occupational Risks and Hazards Exposure, Knowledge of Occupational Health and Safety Practice and Safety Measures among Workers of Sheba Leather Plc, Wukro, Tigray Ethiopia. MOJPH. 2016;4(2). doi: 10.15406/mojph.2016.04.00074

[7] De Camargo C. The postcode lottery of safety: COVID-19 guidance and shortages of personal protective equipment (PPE) for UK police officers. The Police Journal: Theory, Practice and Principles. 2021;95(3):537–61. doi: 10.1177/0032258x211018768

[8] Jerie S. Occupational Risks Associated with Solid Waste Management in the Informal Sector of Gweru, Zimbabwe. J Environ Public Health. 2016;2016:9024160. doi: 10.1155/2016/9024160 27418935 PMC4932156

[9] Yankson IK, Nsiah-Achampong NK, Okyere P, Afukaar F, Otupiri E, Donkor P, et al. On-site personal protective equipment signage and use by road construction workers in Ghana: a comparative study of foreign- and locally-owned companies. BMC Public Health. 2021;21(1):2321. doi: 10.1186/s12889-021-12376-2 34949168 PMC8705104

[10] Mohd Sharif KI. The analysis and health risks of workers in the municipal solid waste landfill in Malaysia. 4th Int Conf Technol Oper Manag. 2016.

[11] Marahatta SB, Katuwl D, Adhikari S, Rijal K. Knowledge on occupational health hazard and safety practices among the municipal solid waste handler. J Manmohan Memorial Inst Health Sci. 2018;3(1):56–72. doi: 10.3126/jmmihs.v3i1.19179

[12] Afolabi FJ, de Beer P, Haafkens JA. Occupational Risk Perception and the Use of Personal Protective Equipment (PPE): A Study Among Informal Automobile Artisans in Osun State, Nigeria. Sage Open. 2021;11(1). doi: 10.1177/2158244021994585

[13] Boadi KO, Kuitunen M. Municipal Solid Waste Management in the Accra Metropolitan Area, Ghana in the Accra Metropolitan Area, Ghana. Environmentalist. 2003.

[14] Ishtiaq M. Book Review Creswell, J. W. (2014). Research Design: Qualitative, Quantitative and Mixed Methods Approaches (4th ed.). Thousand Oaks, CA: Sage. In: English Language Teaching. 2019. p. 40.

[15] Ghana Statistical Service. Projected population by district and sex, Volta Region, 2010, 2015-2020. Accra: Ghana Statistical Service. 2018.

[16] Taherdoost H. Sampling methods in research methodology; how to choose a sampling technique for research. SSRN Electron J. 2016;5:18–27.

[17] Henseler J, Ringle CM, Sarstedt M. A new criterion for assessing discriminant validity in variance-based structural equation modeling. J Acad Mark Sci. 2015;43:115–35.

[18] Ayikoru M, Ddamulira C, R Mutekanga D. Determinants of Employee use of Personal Protective Equipment, the Case of Spedag Interfreight Uganda Limited, Kampala. J Environ Sci Public Health. 2019;03(03). doi: 10.26502/jesph.96120073

[19] Wall JM. Development of a health-belief-model-based instrument to assess worker beliefs about using personal protective equipment. 2009.

[20] Wright T, Adhikari A, Yin J, Vogel R, Smallwood S, Shah G. Issue of compliance with use of personal protective equipment among wastewater workers across the southeast region of the United States. Int J Environ Res Public Health. 2019;16(11):2009. doi: 10.3390/ijerph16112009 31195677 PMC6603999

[21] Kretchy J-P, Dzodzomenyo M, Rheinlӓnder T, Ayi I, Konradsen F, Fobil JN, et al. Exposure, protection and self-reported health problems among solid waste handlers in a Coastal Peri-urban community in Ghana. Int J Public Heal Epidemiol. 2015;4:2326–7291. http://curis.ku.dk/ws/files/161185472/Exposure_protection_and_self_reported_health_problems_among_solid_waste_handlers_in_a_Coastal_Peri_urban_community_in_Ghana.pdf

[22] Islam MS, Siddique AB, Akter R, Tasnim R, Sujan MSH, Ward PR, et al. Knowledge, attitudes and perceptions towards COVID-19 vaccinations: a cross-sectional community survey in Bangladesh. BMC Public Health. 2021;21(1):1851. doi: 10.1186/s12889-021-11880-9 34645399 PMC8513387

[23] Mwangi GM, Despoudi S, Espindola OR, Spanaki K, Papadopoulos T. A planetary boundaries perspective on the sustainability: resilience relationship in the Kenyan tea supply chain. Ann Oper Res. 2022;319(1):661–95. doi: 10.1007/s10479-021-04096-y 34024979 PMC8130987

[24] Das SS. Occupational health problems among door to door solid waste handlers in Surat City, Gujarat. Diss submitted partial fulfilment requirement for award of degree Master Public Health. 2009.

[25] Lissah SY, Ayanore MA, Krugu JK, Aberese-Ako M, Ruiter RAC. “Our Work, Our Health, No One’s Concern”: Domestic Waste Collectors’ Perceptions of Occupational Safety and Self-Reported Health Issues in an Urban Town in Ghana. Int J Environ Res Public Health. 2022;19(11):6539. doi: 10.3390/ijerph19116539 35682123 PMC9180064

[26] Ramayah T, Cheah J, Chuah F, Ting H, Memon MA. Partial least squares structural equation modeling (PLS-SEM) using SmartPLS 3.0: an updated guide and practical guide to statistical analysis. 2nd ed. Kuala Lumpur, Malaysia: Pearson. 2018.

[27] Hair JF, Risher JJ, Sarstedt M, Ringle CM. When to use and how to report the results of PLS-SEM. Eur Bus Rev. 2019;31:2–24.

[28] Mossburg S, Agore A, Nkimbeng M, Commodore-Mensah Y. Occupational Hazards among Healthcare Workers in Africa: A Systematic Review. Ann Glob Health. 2019;85(1):78. doi: 10.5334/aogh.2434 31172728 PMC6634430

[29] Sialubanje C, Massar K, Hamer DH, Ruiter RAC. Personal and environmental predictors of the intention to use maternal healthcare services in Kalomo, Zambia. Health Educ Res. 2014;29(6):1028–40. doi: 10.1093/her/cyu057 25274723

[30] Krugu JK, Mevissen FEF, Debpuur C, Ruiter RAC. Psychosocial Correlates of Condom Use Intentions among Junior High School Students in the Bolgatanga Municipality of Ghana. International Journal of Sexual Health. 2015;28(1):96–110. doi: 10.1080/19317611.2015.1124162

[31] Nyembezi A, Ruiter RAC, van den Borne B, Sifunda S, Funani I, Reddy P. Correlates of consistent condom use among recently initiated and traditionally circumcised men in the rural areas of the Eastern Cape Province, South Africa. BMC Public Health. 2014;14:668. doi: 10.1186/1471-2458-14-668 24975721 PMC4083871

[32] Fishbein M, Ajzen I. Predicting and Changing Behaviour. The Reasoned Action Approach (3rd ed.). New York, NY: Taylor and Francis. 2010.

[33] Bandura A. Health promotion from the perspective of social cognitive theory. Psychol Heal. 1998;13:623–49.

[34] Rosenstock IM. Historical origins of the health belief model. Health Educ Behav. 1974;2:328–35.

[35] Hair JF, Hult GTM, Ringle CM, Sarstedt M. A primer on partial least squares structural equation modeling (PLS-SEM). 2nd ed. United States of America: SAGE Publications, Inc. 2017.

[36] Shmueli G, Sarstedt M, Hair JF, Cheah JH, Ting H, Vaithilingam S. Predictive model assessment in PLS-SEM: guidelines for using PLSpredict. Eur J Mark. 2019;53:2322–47.

[37] Lissah SY, Ayanore MA, Krugu J, Ruiter RAC. Psychosocial Risk, Work-Related Stress, and Job Satisfaction among Domestic Waste Collectors in the Ho Municipality of Ghana: A Phenomenological Study. Int J Environ Res Public Health. 2020;17(8):2903. doi: 10.3390/ijerph17082903 32331457 PMC7215998

[38] Lissah SY, Ayanore MA, Krugu JK, Aberese-Ako M, Ruiter RAC. Managing urban solid waste in Ghana: Perspectives and experiences of municipal waste company managers and supervisors in an urban municipality. PLoS One. 2021;16(3):e0248392. doi: 10.1371/journal.pone.0248392 33705483 PMC7951920

[39] Cochran WG. Sampling techniques. 3rd ed. New York: John Wiley & Sons. 1977.

[40] Ringle CM, Wende S, Becker JM. SmartPLS 3 [Software]. Bönningstedt: SmartPLS GmbH. 2022.

[41] Henseler J, Ringle CM, Sarstedt M. A new criterion for assessing discriminant validity in variance-based structural equation modeling. J Acad Mark Sci. 2015;43.

[42] Sarstedt M, Hair JF Jr, Cheah J-H, Becker J-M, Ringle CM. How to Specify, Estimate, and Validate Higher-Order Constructs in PLS-SEM. Australasian Marketing Journal. 2019;27(3):197–211. doi: 10.1016/j.ausmj.2019.05.003

[43] Cohen P, Cohen J, West S, Aiken LS. Applied multiple regression/correlation analysis for the behavioral sciences third edition copyrighted material. 2003.

[44] Usakli A, Kucukergin KG. Using partial least squares structural equation modeling in hospitality and tourism: do researchers follow practical guidelines?. Int J Contemp Hosp Manag. 2018;30:3462–512.

[45] Stone M. Cross-validatory choice and assessment of statistical predictions. J R Stat Soc. 1974;36:111–33.

[46] Davcik NS. The use and misuse of structural equation modeling in management research: A review and critique. J Adv Manag Res. 2014;11:47–81.

[47] Peng DX, Lai F. Using partial least squares in operations management research: A practical guideline and summary of past research. J of Ops Management. 2012;30(6):467–80. doi: 10.1016/j.jom.2012.06.002

[48] Abdollahzadeh G, Sharifzadeh MS. Predicting farmers’ intention to use PPE for prevent pesticide adverse effects: An examination of the Health Belief Model (HBM). Journal of the Saudi Society of Agricultural Sciences. 2021;20(1):40–7. doi: 10.1016/j.jssas.2020.11.001

[49] Xu S, Zou PXW, Luo H. Impact of attitudinal ambivalence on safety behaviour in construction. Adv Civ Eng. 2018;2018.

[50] Tessema M, Sema W. Utilization of Personal Protective Equipment and Associated Factors among Large-Scale Factory Workers in Debre-Berhan Town, Amhara Region, Ethiopia, 2021. J Environ Public Health. 2022;2022:8439076. doi: 10.1155/2022/8439076 35178097 PMC8847034

