# Supplementary material for: Assessing the socio-cognitive determinants of personal protective equipment uses among domestic waste collectors in the Ho municipality, Ghana: A cross-sectional study
Source: PLoS One. 2025 Nov 4;20(11):e0334542. doi: 10.1371/journal.pone.0334542 (PMC12585055; doi:10.1371/journal.pone.0334542)
Supplement: S1 Appendix — (DOC) [file pone.0334542.s001.doc]

**S1 Appendix. Survey instrument for domestic waste collectors**

Location………………………………….. Date: …………………

Name of interviewer:

Company category: **Company A ( ) Company B ( )**

Time interview started

|  |  |  |  |
| --- | --- | --- | --- |
|  |  |  |  |

Time interview ended

**INSTRUCTIONS**: You are requested to provide information about yourself by indicating with a cross (x) or check (√) or, where applicable, entering a number in the spaces provided. The questionnaire is anonymous, which means it will not be possible to identify you or link any responses to you.

SECTION 1: SOCIO-DEMOGRAPHICS

| **No** | **QUESTIONS AND FILTERS** | **CODING CATEGORIES** |
| --- | --- | --- |
| 1 | What is your age? [*in complete years*] | AGE: 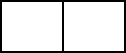 |
| 2 | Gender | Male: …… 1  Female: … 2 |
| 3 | Ethnicity | Ewe ( ) …………………………...1  Ga ( ) …………………………….2  Akan ( ) ………………………….3  Others (Specify) ………………4 |
| 4 | What religion do you practice? | Christianity ( ) ……………………1  Islam ( ) …………………………..2  African Traditional Religion ( ) …..3  Other (specify) ………………..4 |
| 5 | What is your marital status? | Single ( ) …………………………..1  Married ( ) …………………………2  Divorced ( ) ………………………3  Widowed ( ) ………………………4 |
| 6 | What is your level of formal education? | MSLC ( ) ………………................1  Junior High School ( ) ..………....2  Vocational training ( ) ….……….3  Senior High School ( ) .…………4  Tertiary ( ) .……………..…........5  Never been to school ( ) .………..6 |
| 7 | Job role | DWCs (Cleaner, Sweeper, Driver, etc) ( ) … 1  Manager ( ) ………………………….5  Supervisor ( ) ………………………. 6 |
| 8 | Number of years at the workplace: | 1-5 years ( )……………………… 1  6-10 years ( ) ……………………2  11-15 years ( ) ……………………3  16-20 years ( ) ……………………4  over 20 years ( ) ………………… 5 |
|  | **SECTION 2: PERSONAL PROTECTIVE EQUIPMENT USE** | |
|  | **QUESTIONS AND FILTERS** | **CODING CATEGORIES** |
| 9 | Have you participated in any safety training on solid waste management? [If *No*, go to Question 11] | Yes ( ) …………………………….1  No ( ) …………………………… 2 |
| 10 | [If *yes* to Question 9] What type of training on Personal Protective Equipment have you had during your time on the job? (*Tick all that apply*). | i. Familiarization training () …………..1  ii. Basic safety training () ……………...2  iii. Hazard Communication Training () …3  iii. Supervisor safety training () ………...4  iv. Advanced personal protective training (OSHA courses, etc.) () ….……………...5 |
| 11 | How often do you wear personal protective equipment at work? *(Circle the appropriate number)* | Never Rarely Sometimes Very Often Always  1 2 3 4 5 |
| 12 | Which of the following personal protective equipment do you have access to? (*Tick all that apply*) | i. Helmet () …………………………….1  ii. Coveralls () ………………………...2  iii. Safety goggles () …………………..3  iv. Earmuffs/earplugs () ………………..4  v. Gloves () ……………………………5  vi. Wellington boots () …………………6  vii. Safety Shoes (Rubber/Steel etc) ()…7  viii. Respirators/Face mask () …………8  ix. Other (specify) () ………………….9 |
| 13 | Personal protective equipment is ...  i. Important  ii. Uncomfortable  iii. Expensive | Circle a number that best describes your opinion on a 1-5 point scale as shown below:  Strongly Disagree Disagree Undecided Agree Strongly Agree  1 2 3 4 5  Strongly disagree 1 2 3 4 5 Strongly agree  Strongly disagree 1 2 3 4 5 Strongly agree  Strongly disagree 1 2 3 4 5 Strongly agree |
| 14 | When do you use personal protective equipment? [*Tick all that apply*] | i. When on duty? ( )  ii. When a supervisor is around? ( )  iii. When I feel like so? ( )  iv. When it is cold? ( ) |
|  | **SECTION 3: MORBIDITIES ASSOCIATED WITH WASTE COLLECTION** | |
|  | **QUESTIONS AND FILTERS** | **CODING CATEGORIES** |
| 15 | Do you face occupational hazards at your workplace? [*If No, go to Question 17*] | Yes ( ) ……………………………. 1  No ( ) ……………………………..2 |
| 16 | If yes, can you identify some of these exposures at your workplace? [*Tick all that apply*]. | i. Needles () …………………………....1  ii. Vector-borne diseases e.g. mosquitoes.. 2  iii. Abrasions (minor cuts, open wounds, etc.) ().........................3  iv. Respiratory Issues (Dust, Bio-Aerosols, etc) () ………………………….……4  v. Musculoskeletal injuries (Pain in the joints, muscles, and ligaments from repetitive movement () …………………………..5  vi. Burns by steam or hot vapors () ………..6  vii. Slips and Falls () …………………..7  viii. Exposure to excessive noise levels…...8  ix. Discomfort and psychological problems (PPE use, bad smells) () ………………9 |
| 17 | Have you experienced any injuries /diseases in the past 6-12 months? [*If No, go to question 19*] | Yes () …………………………………. 1  No ( ) ………………………………….. 2 |
| 18 | If yes, what type of injuries have you experienced over the past 6-12 months? [*Tick all that apply*] | i. Cutting injury () ……………………….. 1  ii. Puncture wound ( ) ……………………..2  iii. Contusion /Bruise () ……………………3  iv. Cut and Lacerated wound () …………....4  v. Fracture ( ) ……………………………...5  vi. Strain/sprain () ………………………….6  vii. Other (specify) ..………………………7 |
| 19 | In your view, what do you think are the main reasons for injuries and fatalities among domestic waste collectors? [*Tick all that apply*] | i. Lack of visibility around the truck ( )…….1 ii. Insufficient training ( ) …………………..2  iii. Non-observance of safety procedures ( )…3  iv. Nature of work ( ) ………………………..4  v. Improper disposal of waste by residents()...5  vi Lack of proper personal protective equipment ( ) ……………………………..6  vii. Careless overtaking of other vehicles ( )..7  viii. Incentive to work quickly ( ) …………8  ix. Lack of provision of temporary job change due to illness ( )………………… …. …... 9  x. Other (Specify)………………..……. 10 |
| 20 | Have you had any musculoskeletal problems in the past 6-12 months? | Yes () …………………………………….1  No ( ) …………………………………….2 |
| 21 | If yes, which of the following musculoskeletal problems have you experienced at your workplace? [*Tick all that apply*] | i. Weakness in any of your arms, hands, legs, or feet ( ) ………………………………….1  ii. Difficulty moving your arms and legs ( )..2  iii. Pain or stiffness when you lean forward or backward at the waist ( ) ..…………………3  iv. Difficulty bending at your knees ( ) ……4  v. Pain/ Stiffness in Joints ( ) …………….. 5  vi. Neck pain ( ) …………………………..6  vii. Back pain ( ) …..……………………...7  viii. Shoulders pain ( ) …………………….8  ix. Wrist/ hands ( ) ………………………..9  x. Hip pain ( ) ……………………………10  xi. Knee pain ( )………………………….11 |
| 22 | Have you had any eye problem (s) during solid waste collection? | Yes ( ) ……………………………….. 1  No ( ) ………………………………..2 |
| 23 | If yes, which of the listed eye problems have you had during solid waste collection? [*Tick all that apply*] | i. Redness of eyes ( ) …………………...1  ii. Burning eyes ( ) ………………………2  iii. Eye injury ( ) ………………………….3  iv. Other (specify) …………………..…….4 |
| 24 | Have you had any respiratory problems in the past 6-12 months? | Yes ( ) ……………………………….. 1  No ( ) ………………………………...2 |
| 25 | If yes, what kind of respiratory problem (s) have you had in the past 6 - 12 months? (Tick all that apply) | i. Running nose ( ) ……………………1  ii. Dry cough ( ) ………………………2  iii. Cough with phlegm ( ) …………….3  iv. Sneezing ( ) ………………………..4  v. Difficulty in breathing ( ) …………..5  vi. Asthma ( ) …………………………6 |
| 26 | Have you had any skin problem (s) in the past 6- 12 months? | Yes ( ) ……………………………… ..1  No ( ) ……………………………….. .2 |
| 27 | If yes, what kind of skin problem (s) have you had during the past 6-12 months? (*Tick all that apply*) | i. Skin rashes ( ) ……………………….1  ii. Dermatitis ( ) ………………………...2  iii. Allergic rash ( )……………………..3  iv. Skin irritation ( ) …………………….4 |
| 28 | Have you had any gastrointestinal problems in the past 6-12 months? | Yes () …………………………………. 1  No ( ) ………………………………….. 2 |
| 29 | If yes, What kind of gastrointestinal problem (s) have had in the past 6 - 12 months? [Tick all that apply) | i. Vomiting () ………………………….1  ii. Diarrhoea ()………………………….2  iii. Dysentery ()…………………………3  iv. Burning Pain in the abdomen ()…………4  v. Any others, please specify ()………….5 |
|  | **SECTION 4: HAZARDS ASSOCIATED WITH WASTE COLLECTION** | |
|  | **QUESTIONS AND FILTERS** | **CODING CATEGORIES** |
|  | In your opinion, how often do you come across the following hazards in performing your job? | Circle a number that best describes your opinion on a 1-5 point scale as shown below:  Never Rarely Sometimes Very Often Always  1 2 3 4 5 |
| 30 | Cuts and pricks from sharp objects (such as pieces of glass, razor blades, scrap metal, and needles) | Never 1 2 3 4 5 Always |
| 31 | Harsh weather conditions (exposure to sun, wind, and rain) | Never 1 2 3 4 5 Always |
| 32 | Bites by rodents (such as mice etc.) | Never 1 2 3 4 5 Always |
| 33 | Infections transmitted by insects | Never 1 2 3 4 5 Always |
| 34 | Exposure to excreta/feces | Never 1 2 3 4 5 Always |
| 35 | Stepping on sharp objects | Never 1 2 3 4 5 Always |
| 36 | Exposure to harmful chemicals | Never 1 2 3 4 5 Always |
|  | **SECTION 5: FACTORS THAT CAN EXPOSE WASTE COLLECTORS TO OCCUPATIONAL HAZARDS** | |
|  | **QUESTIONS AND FILTERS** | **CODING CATEGORIES** |
|  | In your opinion, to what extent can the following factors expose domestic waste collectors to occupational health and safety hazards | Circle a number that best describes your opinion on a 1-5 point scale as shown below:  Strongly Disagree Disagree Undecided Agree Strongly Agree  1 2 3 4 5 |
| 37 | Low-level literacy among workers | Strongly disagree 1 2 3 4 5 Strongly agree |
| 38 | Negligence on the part of waste workers | Strongly disagree 1 2 3 4 5 Strongly agree |
| 39 | Lack of health and safety awareness | Strongly disagree 1 2 3 4 5 Strongly agree |
| 40 | Negative attitudes and perceptions of workers toward health and safety | Strongly disagree 1 2 3 4 5 Strongly agree |
| 41 | Lack of adequate resources to manage workplace health and safety | Strongly disagree 1 2 3 4 5 Strongly agree |
| 42 | Lack of top management support in the management of the health and safety of workers | Strongly disagree 1 2 3 4 5 Strongly agree |
| 43 | Lack of health and safety training | Strongly disagree 1 2 3 4 5 Strongly agree |
| 44 | Inadequate personal and protective equipment | Strongly disagree 1 2 3 4 5 Strongly agree |
| 45 | Poor maintenance of personal protective gear | Strongly disagree 1 2 3 4 5 Strongly agree |
|  | **SECTION 6 : HBM/RAA QUESTIONNAIRE** | |
|  | **QUESTIONS AND FILTERS** | **CODING CATEGORIES** |
|  | The questionnaire you are about to fill out measures views on predictive factors for personal protective equipment use and health morbidities among waste collectors | Circle a number that best describes your opinion on a 1-5 point scale as shown below:  Strongly Disagree Disagree Undecided Agree Strongly Agree  1 2 3 4 5 |
|  | **Perceived susceptibility (HBM)** |  |
| 46 | My chances of being injured in my workplace are high. | Strongly disagree 1 2 3 4 5 Strongly agree |
| 47 | I am worried about getting an occupational illness. | Strongly disagree 1 2 3 4 5 Strongly agree |
| 48 | I feel that I have a good chance of getting injured at my job | Strongly disagree 1 2 3 4 5 Strongly agree |
| 49 | My exposure to occupational noise could lead to an illness. | Strongly disagree 1 2 3 4 5 Strongly agree |
|  | **Perceived severity** |  |
| 50 | If I am injured in my workplace, I can suffer long-standing problems | Strongly disagree 1 2 3 4 5 Strongly agree |
| 51 | If I develop an occupational illness, my job would be in jeopardy. | Strongly disagree 1 2 3 4 5 Strongly agree |
| 52 | Problems I would experience from an occupational illness would last a long time | Strongly disagree 1 2 3 4 5 Strongly agree |
| 53 | An occupational illness will not lead to permanent changes in my health. | Strongly disagree 1 2 3 4 5 Strongly agree |
| 54 | My financial security would be endangered if I develop an occupational illness. | Strongly disagree 1 2 3 4 5 Strongly agree |
| 55 | I think if I develop an occupational disease, I might die prematurely. | Strongly disagree 1 2 3 4 5 Strongly agree |
| 56 | 1 am afraid to even think about getting an occupational illness. | Strongly disagree 1 2 3 4 5 Strongly agree |
|  | **Attitude toward personal protective equipment use** |  |
| 57 | I feel that taking personal protective equipment home is harmful. | Strongly disagree 1 2 3 4 5 Strongly agree |
| 58 | I believe that compliance to the standard operating procedures in wearing personal protective equipment (should be made compulsory) (is important) | Strongly disagree 1 2 3 4 5 Strongly agree |
| 59 | I feel that wearing personal protective equipment is important | Strongly disagree 1 2 3 4 5 Strongly agree |
|  | **Behavioral Beliefs of DWCs on personal protective equipment** |  |
| 60 | The available personal protective equipment is of standard quality. | Strongly disagree 1 2 3 4 5 Strongly agree |
| 61 | Only some special health workers require personal protective equipment | Strongly disagree 1 2 3 4 5 Strongly agree |
| 62 | My present level of knowledge of personal protective equipment is adequate. | Strongly disagree 1 2 3 4 5 Strongly agree |
| 63 | The available personal protective equipment is effective in preventing infectious diseases. | Strongly disagree 1 2 3 4 5 Strongly agree |
|  | **Subjective norm** |  |
| 64 | My co-workers believe that I should wear personal protective equipment | Strongly disagree 1 2 3 4 5 Strongly agree |
| 65 | My workplace supports me in wearing personal protective equipment. | Strongly disagree 1 2 3 4 5 Strongly agree |
| 66 | My family believes that I should wear personal protective equipment | Strongly disagree 1 2 3 4 5 Strongly agree |
| 67 | My friends and neighbors believe that I should wear personal protective equipment | Strongly disagree 1 2 3 4 5 Strongly agree |
|  | **Perceived benefits** |  |
| 68 | Wearing personal protective equipment will prevent future health problems for me | Strongly disagree 1 2 3 4 5 Strongly agree |
| 69 | Personal protective equipment prevents the kind of risks I face from being exposed to in the jobs | Strongly disagree 1 2 3 4 5 Strongly agree |
| 70 | I do not worry about getting an occupational illness when I use personal protective equipment | Strongly disagree 1 2 3 4 5 Strongly agree |
| 71 | I benefit by wearing personal protective equipment | Strongly disagree 1 2 3 4 5 Strongly agree |
| 72 | Wearing personal protective equipment decreases the risk of being injured at the workplace | Strongly disagree 1 2 3 4 5 Strongly agree |
|  | **Perceived barriers/ Perceived behavioral control** |  |
| 73 | Wearing personal protective equipment is uncomfortable | Strongly disagree 1 2 3 4 5 Strongly agree |
| 74 | Personal protective equipment interferes with my ability to do my job | Strongly disagree 1 2 3 4 5 Strongly agree |
| 75 | Wearing personal protective equipment is just an inconvenience for me. | Strongly disagree 1 2 3 4 5 Strongly agree |
| 76 | Personal protective equipment is not always available to me. | Strongly disagree 1 2 3 4 5 Strongly agree |
| 77 | My coworkers would make fun of me for wearing personal protective equipment | Strongly disagree 1 2 3 4 5 Strongly agree |
| 78 | My supervisor seldom wears personal protective equipment when required. | Strongly disagree 1 2 3 4 5 Strongly agree |
| 79 | My supervisor is aware of my compliance with personal protective equipment guidelines | Strongly disagree 1 2 3 4 5 Strongly agree |
| 80 | I often wear personal protective equipment while at work | Strongly disagree 1 2 3 4 5 Strongly agree |
|  | **Behavior Intention** |  |
| 81 | I would need to develop a new habit for wearing personal protective equipment. | Strongly disagree 1 2 3 4 5 Strongly agree |
| 82 | I have planned to wear personal protective equipment regularly | Strongly disagree 1 2 3 4 5 Strongly agree |
| 83 | I am likely to use personal protective equipment during working in the next six months | Strongly disagree 1 2 3 4 5 Strongly agree |
| 84 | I am likely to use personal protective equipment the next time I am working | Strongly disagree 1 2 3 4 5 Strongly agree |
| 85 | I will put on the highest level of personal protective equipment when the need arises. | Strongly disagree 1 2 3 4 5 Strongly agree |
| 86 | I will use the personal protective equipment provided by the company in the workplace. | Strongly disagree 1 2 3 4 5 Strongly agree |
|  | **Cues to action** |  |
| 87 | My supervisor checking up on me would improve my attitude toward the use of personal protective equipment | Strongly disagree 1 2 3 4 5 Strongly agree |
| 88 | The fact that OSHA and/or my employer could fine me helps me to wear personal protective equipment | Strongly disagree 1 2 3 4 5 Strongly agree |
| 89 | Posters in my workplace serve as important reminders to wear personal protective equipment. | Strongly disagree 1 2 3 4 5 Strongly agree |
| 90 | The threat of disciplinary action is an important factor in ensuring I wear personal protective equipment | Strongly disagree 1 2 3 4 5 Strongly agree |
| 91 | Having personal protective equipment at the location of the hazard is critical to making sure I wear it | Strongly disagree 1 2 3 4 5 Strongly agree |
| 92 | If I see my workmates wearing personal protective equipment, it reminds me to use it. | Strongly disagree 1 2 3 4 5 Strongly agree |
| 93 | Regular and frequent education on the importance of personal protective equipment serves to improve how often I wear it | Strongly disagree 1 2 3 4 5 Strongly agree |
| 94 | My supervisor sets the example of wearing personal protective equipment when exposed | Strongly disagree 1 2 3 4 5 Strongly agree |
| 95 | My co-workers who wear personal protective equipment often remind me to use mine | Strongly disagree 1 2 3 4 5 Strongly agree |
|  | **Self-efficacy** |  |
| 96 | I am confident that I will remember to use personal protective equipment when I am exposed to hazards at work | Strongly disagree 1 2 3 4 5 Strongly agree |
| 97 | I am confident that I can obtain the proper personal protective equipment when I am exposed to hazards at work | Strongly disagree 1 2 3 4 5 Strongly agree |
| 98 | I am confident that my job performance will not be adversely impacted by wearing personal protective equipment | Strongly disagree 1 2 3 4 5 Strongly agree |
| 99 | I am confident that the personal protective equipment I use when I am exposed to hazards at work is the proper equipment to protect me | Strongly disagree 1 2 3 4 5 Strongly agree |
| 100 | I am confident that wearing proper personal protective equipment throughout my job will help prevent me from getting an occupational illness | Strongly disagree 1 2 3 4 5 Strongly agree |
| 101 | 1 can prevent an occupational illness | Strongly disagree 1 2 3 4 5 Strongly agree |
|  | **SECTION 7 MANAGERS/ SUPERVISORS** | Circle a number that best describes your opinion on a 1-5 point scale as shown below:  1 2 3 4 5  Never Rarely Seldom Often Very often |
| 102 | How often do you enforce wearing personal protective equipment? | Never 1 2 3 4 5 Very often |
| 103 | How often do you set the example of wearing personal protective equipment when being exposed to hazards | Never 1 2 3 4 5 Very often |
| 104 | How often are you aware of your employees’ compliance to personal protective equipment? | Never 1 2 3 4 5 Very often |
| 105 | How often do you threaten disciplinary action if personal protective equipment regulations are not followed? | Never 1 2 3 4 5 Very often |
| 106 | How often do you ensure that personal protective equipment is available for your employees? | Never 1 2 3 4 5 Very often |
| 107 | How often do you provide regular and frequent education on the importance of personal protective equipment? | Never 1 2 3 4 5 Very often |

**Thank You for Your Time**
